# *In situ* vaccination caused by diverse irradiation-driven cell death programs

**DOI:** 10.7150/thno.86004

**Published:** 2024-01-12

**Authors:** Yijun Wang, Yan Li, Yuxin Yang, Michelle Swift, Zhenyu Zhang, Shuhui Wu, Yajie Sun, Kunyu Yang

**Affiliations:** 1 Cancer Center, Union Hospital, Tongji Medical College, Huazhong University of Science and Technology, Wuhan 430022, China.; 2 Hubei Key Laboratory of Precision Radiation Oncology, Wuhan 430048, China.; 3 University of Southern California, Department of Biochemistry and Molecular Medicine.; 4 Department of Radiation Oncology, Dana-Farber Cancer Institute, Boston, MA, 02215, USA.; 5 Department of Biostatistics, Fielding School of Public Health, University of California, Los Angeles, California 90095-1772, USA.

**Keywords:** Cell death programs, *in situ* vaccine, irradiation, immunotherapy

## Abstract

Interest surrounding the effect of irradiation on immune activation has exponentially grown within the last decade. This includes work regarding mechanisms of the abscopal effect and the success achieved by combination of radiotherapy and immunotherapy. It is hypothesized that irradiation triggers the immune system to eliminate tumors by inducing tumor cells immunogenic cell death (ICD) in tumor cells. Activation of the ICD pathways can be exploited as an *in situ* vaccine. In this review, we provide fundamental knowledge of various forms of ICD caused by irradiation, describe the relationship between various cell death pathways and the immune activation effect driven by irradiation, and focus on the therapeutic value of exploiting these cell death programs in the context of irradiation. Furthermore, we summarize the immunomodulatory effect of different cell death programs on combinative radiotherapy and immunotherapy. In brief, differences in cell death programs significantly impact the irradiation-induced immune activation effect. Evaluating the transition between them will provide clues to develop new strategies for radiotherapy and its combination with immunotherapy.

## Introduction

The prevailing consensus posits that irradiation instigates tumor cell death by inflicting DNA damage, primarily amplifying the overwhelming amount of DNA double-strand breaks (DSBs) 1. More precisely, the etiology of DNA damage and ensuing tumor cell death is often ascribed to the four tenets of radiobiology, collectively known as the “4R” theory, which includes: (i) repair of sublethal damage, (ii) redistribution within the cell cycle, (iii) reoxygenation of tumor cells, and (iv) repopulation of cells in tumor tissue 2. Consequently, the effectiveness of each segment within the “4R” paradigm can modulate the radiosensitivity of tumor cells. *In vivo*, however, the situation is considerably more intricate. The radiosensitivity of tumors hinges on the complex interplay between the intrinsic sensitivity of the tumor cells and that of the tumor microenvironment. The contribution of the tumor microenvironment in the context of the host's immunity to radiotherapy efficacy remains relatively underexplored.

A contemporary hypothesis as to how radiotherapy eliminates tumors is that it not only directly kills tumor cells, but also contributes to releasing tumor-associated antigens (TAAs) and damage-associated molecular patterns (DAMPs) to activate the immune system, which later mediates anti-tumor immunity, now considered as “*in situ* vaccine” 3-5. Upon further exploration, it is clear that beyond the release of DAMPs and TAAs during tumor cell death, irradiation can initiate the *in situ* vaccine by promoting the release of pro-inflammatory cytokines, activating inherent immune signaling pathways, and presenting neoantigens. The observed abscopal effect offers compelling evidence of the immune system's contribution to radiotherapy's therapeutic efficacy 6, 7. Moreover, the augmented therapeutic results observed when merging immunotherapy with radiotherapy, compared to standalone immunotherapy, underscores the non-negligible immunomodulatory role of irradiation 8.

Given the demonstrated significance of the immune system in the anti-tumor effects of radiation, current research seeks to enhance anti-tumor immune responses *via* radiotherapy. From the immunological perspective, cellular death programs are classified into immunogenic cell death (ICD) and non-ICD categories 9. ICD involves modifications to the tumor cell surface and the release of immunogenic mediators that stimulate dendritic cells (DCs) to present tumor-associated antigens (TAAs) or neoantigens to T cells, resulting in sustained anti-tumor immunity 10. Since different forms of cell death can be elicited by irradiation, functioning as an “*in situ* tumor vaccine”, it is crucial to discern the modes of cell death programs driven by irradiation and their respective impacts on immune activities. This review primarily explores the mechanisms of multiple cell death programs initiated by irradiation, and how their regulation can augment anti-tumor immunity.

## The Brief Depicting of Radiotherapy

Radiotherapy stands as a cornerstone treatment for malignant solid tumors, with an estimated 70% of cancer patients requiring its application and 40% of tumors being potentially curable through its utilization 11. The origin of contemporary radiotherapy can be traced back to the late 19th century, punctuated by pivotal milestones: (i) Roentgen's unveiling of X-rays in 1895; (ii) Becquerel's identification of natural radioactivity in 1896; and (iii) the Curies' extraction of radium in 1898. By 1896, therapeutic radiation treatments were initiated in countries like France, the USA, and Sweden. Nevertheless, the advent of side effects such as skin damage, nausea and fatigue highlighted the imperative to optimize radiation doses and fractions, all while improving radiation protection. Over time, basic kilovolt radiotherapy evolved into advanced computer-assisted precision techniques, now progressing towards proton-mediated radiotherapy 12, 13.

It is generally recognized that the efficacy of ionizing radiation comes from its targeting of DNA. Ionizing radiation can induce DNA damage through both direct and indirect effects. Direct effect, as the name implies, refers to the direct attack of radiating electron beams or photon beams on DNA molecules, thereby causing direct DNA damage; while the indirect effect refers to electron or photon beams colliding with intracellular water molecules and producing charged groups (mainly free radicals ·OH), which then attack DNA molecules and proteins. Both direct and indirect effects contribute to the breakage of chemical bonds in DNA molecules, resulting in the loss of a nucleotide base, an entire codon sequence, or even causing the breakage of the pentose phosphate skeleton, involving one or two DNA strands, namely DNA single strand breaks (SSBs) or DNA double strand breaks (DSBs). SSBs typically occur at lower radiation doses, while DSBs usually manifest at higher doses. Contrary to SSBs, which infrequently lead to cell death due to their general repairability using complementary strands as templates, DSBs often culminate in chromatin fragmentation and consequent cell death due to their substantial frequency 14.

In order to reduce genomic instability caused by DNA damage and maintain survival after ionizing radiation, tumor cells initiate DNA damage response and deploy DNA repair proteins to address the damaged DNA 15. DNA damage is perceived by the ataxia-telangiectasia mutant gene (ATM) and the ataxia-telangiectasia Rad3-related protein (ATR), which activates the downstream checkpoint kinase CHEK1 and CHEK2, subsequently phosphorylating the p53 gene, which stops the cell cycle and initiates the DNA damage response 16. During the process, two types of DNA damage repair mechanisms take precedence: non-homologous end joining (NHEJ) and homologous recombination (HR). NHEJ has a high probability of error and can be active throughout the cell cycle, while HR has higher fidelity whereas it requires an undamaged sister chromatid as a repair template, thus, it is operational only during the S phase and late G2 phase of the cell cycle 17, 18. Irreparable DNA damage is fatal to tumor cells and initiates cell clearance processes-the following cell death programs 19.

## Advances in Radiotherapy Technology Pave the Way for Enhanced Clinical Outcomes

The landscape of radiation therapy techniques has progressed from the traditional two-dimensional (2D) radiation therapy (2D-RT) to three-dimensional conformal radiation therapy (3D-CRT), followed by intensity-modulated radiation therapy (IMRT) and proton-based radiation therapy. Alongside these advancements, enhanced dosimetric characterization has resulted in improved local area control and survival rates. Currently, IMRT stands as the most prevalent radiation therapy technique in clinics.

The technique of 3D-CRT enables imaging scans to generate detailed 3D models of the tumor and the adjacent tissue, facilitating clinicians in customizing the radiation beams to conform to the tumor's shape, thereby reducing exposure to healthy tissue. This technique is employed for a variety of cancers, covering prostate, breast, and lung cancers 20-23. IMRT represents an advanced form of 3D-CRT, offering the capability to modulate the intensity of radiation beams. This feature enables an even more precise sculpting of radiation dose to target tumors, consequently enhancing therapeutic effectiveness and mitigating toxicity 24. It is utilized in scenarios where the tumor is in proximity to critical structures, for instance in cases of head and neck, prostate, and central nervous system cancers 25. Taking nasopharyngeal carcinoma as an illustration, the application of IMRT has led to a noteworthy reduction in the 5-year incidence of local failure among newly diagnosed and non-metastatic patients, now standing at 7.4% 26. In a meta-analysis encompassing over 3570 patients with nasopharyngeal carcinoma, it turned out that IMRT was strongly correlated with superior 5-year locoregional control (odds ratio 1.9) and overall survival (odds ratio 1.51) when compared to 2D-RT or 3D-CRT 27. Another notable investigation showcasing the advantages of IMRT is the study RTOG 0617 in lung cancer 28. This study juxtaposed the use of standard-dose and high-dose conformal radiation therapy, in addition to the optional use of IMRT in patients diagnosed with stage III non-small cell lung cancer. Although it concluded that escalating the radiation dose was not able to enhance patient survival rates, it was discerned that IMRT was linked with decreased instances of severe pneumonitis and reduced cardiac doses in comparison to 3D-CRT. Proton-based radiation therapy, in contrast to X-ray-based approaches, harnesses protons in cancer treatment. Protons possess a unique property known as the “Bragg Peak”, enabling them to dispense the majority of their energy at a specific depth, thereby minimizing damage to the adjacent tissue. This radiation method is frequently utilized for tumors situated near sensitive regions such as the brain, spinal cord, and eye, or in pediatric patients 29.

Advances in radiotherapy technology have enabled not only precise dose control within the radiation field and minimized radiation spillage but also facilitated stronger tumor eradication. A multitude of clinical trial results underscore the direct association between the advancement of radiotherapy techniques and improved patient outcomes.

## Post Irradiation Effects Driven by Cell Death: *In situ* Vaccination

The impact of irradiation on cell death programs is widespread. On the one hand, in the primary stage following radiotherapy, tumor cells predominantly initiate stress-response programs to mitigate the damage *via* a number of signal transduction pathways 30. The responding process involves modifications in the expression of various genes, which are dependent on the genetic background of the host, including p53 status, as well as the radiation fractions or doses received 31-34. Among the up-regulated genes are those responsible for regulating the expression of cytokines, chemokines, growth factors and cell surface receptors crucial for mediating the interaction between the tumor and the immune system 35, 36. Of these, the surface molecules closely associated with the activation of anti-tumor immunity belong to the MHC-I molecules and PD-L1 molecules. The upregulation of the former following radiotherapy enhances the specific recognition and subsequent killing by T cells, while the latter provides targets for immune checkpoint inhibitors, establishing the groundwork for the synergistic effect of radiotherapy and immunotherapy. On the other hand, the types of cell death following radiation exhibit considerable variability, spanning from apoptosis, necroptosis to pyroptosis and ferroptosis. Significantly, these various forms of cell death induced by irradiation predominantly exhibit immunogenic characteristics. Such immunogenic features of irradiation-driven cell death are bolstered by several pivotal factors including the release of High-Mobility Group Protein B1 (HMGB1), the emission of adenosine-triphosphate (ATP), and the exposure of Calreticulin (CRT) on the tumor cell surface 37-42. Simultaneously, irradiated tumor cell-derived extracellular vesicles (EVs) or microparticles (MPs) also display immunomodulatory effects, with research conducted by Wan C et al. proved that the bystander effect of radiotherapy was primarily mediated by such microparticles 43. These agents are recognized for promoting the maturation and activation of dendritic cells (DCs), the principled antigen-presenting cells, thereby facilitating the cross-priming of CD8^+^ T cell-mediated adaptive immunity. Succinctly put, radiation acts as a substantial modifier of the tumor microenvironment, provoking ICD of tumor cells and alterations in tumor immunogenicity 44. Following the activation of the systemic anti-tumor immunity, localized radiation impacts not only the targeted sites but also influences regions beyond the irradiated field, functioning as an “*in situ* tumor vaccine” which inhibits tumor growth both locally and systemically 45, 46.

The abscopal effect provides compelling evidence of the efficacy of *in situ* vaccines triggered by ionizing radiation, particularly in collaboration with the host's systemic immunity. Initially identified by Mole RH et al. in 1953, this phenomenon illustrates how untreated tumors can undergo regression in parallel with those receiving localized treatment 47. Subsequently, in 2004, Formenti and Demaria theorized that irradiated tumor cells might liberate specific factors that stimulate the immune system This process mirrors classical immunity, involving antigen presentation DCs and the activation and proliferation of subsequent effector T cells 48. The phenomenon then captured broader interest in 2012 when a melanoma patient, subjected to both immunotherapy and radiotherapy, exhibited regression in the primary tumor and its untreated metastases 49. Since then, the intricate interplay among radiotherapy, host immunity, and the abscopal effect has ascended as a pivotal topic.

The impact of ionizing radiation on *in situ* vaccines bridges two distinct yet partially overlapping fields, prompting a reconsideration of classical damage responses and the resultant multiple cell death pathways occurring at the irradiated site within the context of systemic immunity 7. Contemporary studies now imply a crosstalk between immunogenic cell death pathways and immune system *via* irradiation-driven* in situ* vaccination, suggesting that irradiation perpetually initiates immunogenic cell death programs and the presentation of antigens for anti-tumor immunity. The impact of *in situ* vaccines on activating anti-tumor immune responses may be long-lasting, persisting beyond the conclusion of radiotherapy. In tumor cells that are susceptible to radiation, immunogenic cell death is more likely to occur and the neoantigens generated during this process have not been previously recognized by the immune system. Consequently, the ensuing immune responses are robust and prolonged. Although tumor cells with heightened DNA damage repair capability may not invariably undergo cell death, radiation exposure contributes to their genomic instability, making them more susceptible to genetic mutations. This accumulation of mutations leads to the generation of neoantigens, effectively creating an *in situ* tumor vaccine. Furthermore, the irradiation-induced cytokine cascade can intensify immune activation. Specifically, the release of certain cytokines early after radiotherapy can stimulate the production of pro-inflammatory cytokines, resulting in a sustained immune response.

## The Various Cell Death Programs Driven by Irradiation

Traditional radiobiology posits that ionizing radiation exterminates tumor cells by targeting DNA molecules. However, as insights into the "*in situ* vaccine" impact of irradiation emerge, this conventional theory cannot fully encompass the intricate interplay between various cell death pathways and immune responses. Hence, discerning how systemic immunity adjusts to different cell death modalities becomes crucial. Such understanding will enable clinicians to better grasp the synergistic mechanics of radiotherapy and immunotherapy, paving the way for refined clinical practice.

### Irradiation and Apoptosis

Apoptosis, coined by Kerr and Wyllie in 1972, was defined as an energy-dependent cell suicide accompanied by cell contraction, membrane bledding, and formation of apoptotic bodies 50-53. Due to its regulation by related genes, apoptosis is considered a form of programmed cell death. The occurrence of apoptosis is mainly initiated by two distinct pathways, intrinsic and extrinsic. A few years after this discovery, the radiobiologists discovered that tumor cells exhibit an apoptotic phenotype after irradiation. Currently, apoptosis is the most predominant form of cell death driven by irradiation.

In tumor cells, ionizing radiation triggers a chain of irreversible disturbances. The most prevalent among these perturbations are the apoptosis processes mediated by two distinct pathways. In the intrinsic pathway, mitochondrial modifications governed by the Bcl-2 family proteins serve as the primary initiators. Upon exposure to ionizing radiation, the equilibrium between pro-apoptotic and anti-apoptotic factors is disrupted. Here, pro-apoptotic agents like Bcl-2 associated X-protein (BAX), Bcl-2 antagonist/killer 1 (BAK1), and p53 upregulated modulator of apoptosis (PUMA) surpass the anti-apoptotic agents, leading to a surge in Mitochondrial Outer Membrane Permeabilization (MOMP). A disruption in the mitochondrial outer membrane facilitates the release of Cytochrome C, which then collaborates with Apaf-1 and ATP to activate pro-caspase-9, subsequently leading to caspase-9 activation 54-57. Caspase-3, caspase-6, and caspase-7, the downstream executioners, are then activated by caspase-9 to commence apoptosis. Additionally, ionizing radiation bolsters the union of death ligands and death receptors, triggering extrinsic apoptosis. The interaction of death ligands with death receptors transforms pro-caspase-8 into caspase-8, spurring the activation of the Death-Inducing Signaling Complex (DISC), as well as complexes I and II. These complexes serve as molecular platforms to further drive extrinsic apoptosis 58-60. Notably, radiation exposure also elevates the expression of death receptors, including FAS, TNF receptors, and TRAIL receptors, contributing to the initiation of death programs as well 61-63.

In their seminal research on apoptosis, Kerr highlighted that even though a significant number of cells underwent apoptosis, no inflammatory response was detected. This absence of inflammation can be ascribed to the maintenance of cell membrane integrity and the swift phagocytic engulfment by phagocytes 50. The “integrity” of the cell membrane and the rapid phagocytosis of dying cells and their debris, which was later referred to as “efferocytosis”, prevents the release of cell contents, further prohibits inflammation to activate systemic immune responses 64. According to the statements, apoptosis is considered to be non-immunogenic.

### Irradiation and Necroptosis

Necroptosis, introduced by Degterev in 2005, is another form of ICD, triggered by irradiation 65. Distinct from other forms of programmed cell death, the occurrence of necroptosis is independent of caspase protease activity. Instead, it requires pivotal executor receptor interacting serine/threonine kinase 3 (RIPK3) and its phosphorylated substrate mixed-lineage kinase domain-like (MLKL) 66. The phosphorylation event of MLKL produces pore complexes at the plasma membrane, leading to cell swelling, membrane rupture, and DAMPs secretion to trigger inflammatory responses 67. Necroptosis is a cellular self-destruction program activated as an alternative when apoptosis is blocked 68.

Death receptors (DRs)-including tumor necrosis factor receptor 1 (TNFR1), FAS, DR4 and DR5-perceive signals from extracellular stimuli to initiate necroptosis. Upon activation, these receptors engage with the complex of proteins comprising TNFR-associated death domain protein (TRADD), TNFR-associated factor 2 (TRAF2), and cellular inhibitor of apoptosis protein 1 and 2 (cIAP1/2). Within Complex I, the ubiquitination of Receptor-Interacting Protein kinases 1 (RIPK1) is promoted, a crucial step for NF-κB activation to ensure survival 69, 70. Concurrently, if the ubiquitination of RIPK1 is obstructed by cylindromatosis (CYLD), Complex I can effectively transit to Complex II, constituted by RIPK1, Fas Associated *Via* Death Domain (FADD), and caspase-8. When caspase-8 is inhibited or defective, Complex II binds RIPK3 and activates it through phosphorylation 71-74. Phosphorylated RIPK3 subsequently phosphorylates mixed-lineage kinase domain-like protein (MLKL) 75. The oligomerized MLKL subsequently forms pore complexes that can translocate to the plasma membrane 76-78. This permeabilization of the plasma membrane, instigated by MLKL, results in the influx of Ca^2+^ or Na^+^ ions and the direct formation of pore channels, facilitating the release of DAMPs such as mitochondrial DNA (mtDNA), HMGB1, IL33, IL-1α, and ATP 79, 80. Therefore, the process of necroptosis is pro-inflammatory by activating inflammatory signals and the immune response 81.

In 2011, Nehs MA et al. elucidated that necroptosis plays a critical role in thyroid interstitial carcinoma and adrenocortical carcinoma cell death post-irradiation 82. The induction of necroptosis in tumor cells through irradiation hinges on the radiation dose, fractionation schedule, and RIPK3 expression level. In non-small cell lung carcinoma (NSCLC), cells characterized by low RIPK3 expression exhibit a dual activation of both apoptosis and necroptosis, with the mode being dose-dependent. With elevated RIPK3 expression, cells tend to preferentially undergo necroptosis when exposed to ablative hypo-fractionated radiation therapy (HFRT) with doses ≥ 10 Gy/fraction 83. Conversely, cells exhibiting negligible RIPK3 expression, such as A549 and H460, are less likely to undergo irradiation-induced necroptosis 83. Additionally, in colorectal cancer, the combination of radiotherapy with heat therapy can instigate necroptosis 84. Furthermore, within glioblastoma cases, high radiation doses impede caspase-8 activation, culminating in the formation of necrosomes and thus prompting necroptosis. However, lower radiation doses coupled with active caspase-8 predominantly induce apoptosis 85. Interestingly, the application of the RIPK1 inhibitor, Nec-1, can maintain cell survival even with escalating radiotherapy doses, underlining the substantial role of necroptosis. Necroptosis is also observed in B16-F10 melanoma cells when pan-caspase inhibitors have been demonstrated to shift irradiation-driven cell death from apoptosis to a necroptotic response 86. In addition, irradiation-induced interferon-γ (IFN-γ) has been documented to mediate necroptosis in tumor cells lacking caspase-8, a trait that frequently appears in intensely immune-surveilled tumors 87. The mediation is achieved *via* the promotion of phosphatidylserine exposure.

### Irradiation and Pyroptosis

Initially put forward by Brennan MA et al. and Cookson BT et al. in 2001, pyroptosis, incited by inflammasome activation, represents an inflammatory form of cell death distinct from apoptosis 88. It is characterized by the continuous enlargement of cells until membrane rupture, leading to the release of cell contents which then causes a strong inflammatory response 89, 90. Accordingly, pyroptosis is viewed as a form of ICD.

According to The Nomenclature Committee on Cell Death, the initial definition of pyroptosis referred to inflammatory cell death mediated by caspase-1 activation 91. However, in 2015, Shao F et al. and their colleagues revealed the correlation between inflammatory caspases and Gasdermin family protein (GSDM-F) during pyroptosis occurrence, promoting the comprehensive understanding of pyroptosis 92. Currently, the mechanism of pyroptosis involves activated caspases such as caspase-1, caspase-4, caspase-5, and caspase-11 cleave GSDMs proteins at their N-terminus, which subsequently translocate to the cell membrane and produce pores, resulting in differences in osmotic pressure between intracellular and extracellular, followed by enlargement of the membrane until cell membrane rupture 93, 94. Certain caspases associated with apoptosis, such as caspase-3 and caspase-8, also participate in pyroptosis through a cascade reaction involving caspase-8 and caspase-3 that cleave GSDME 95, 96. With the stimulation of TNF-α, apoptosis dependent caspase-8 can directly cleave GSDMD, triggering pyroptosis 97. Granzyme B, a serine protease that is released by cytotoxic lymphocytes and natural killer cells, can cleave GSDME, activating caspase-independent pyroptosis 98, 99. Similarly, caspase-1 or granzyme A participates in cleaving GSDMB to trigger pyroptosis 100, 101. However, the relationship between GSDMA or GSDMC and pyroptosis still remains unknown 102.

The pathways of pyroptosis are commonly divided into two types: the canonical inflammasome activation pathway and the non-canonical inflammasome activation pathway. The canonical inflammasome activation pathway implies that inflammasomes are indispensable. As protein complexes, inflammasomes are composed of sensor proteins, apoptosis-associated speck-like protein containing a CARD (ASC) and downstream caspases. Sensor proteins such as members of the NOD-like receptor family members (NLRP) and the PYHIN family members absent in melanoma 2 (AIM2), play a critical role in detecting extracellular or intracellular stimuli 103-106. For instance, AIM2 is mainly activated by detecting cytoplasmic double-stranded DNA 107, 108. NOD-like receptor family members NLRC4 is activated only by specific PAMPs whereas NLRP3 is much more “tolerant” and responds to viruses, cellular components such as ATP, cathepsin B, endoplasmic reticulum stress and excessive ROS 109, 110. Activated sensor proteins enlist and activate caspase-1 through ASC, leading to the cleavage of the GSDMD N-terminal as well as pro-IL-1β and pro-IL-18, thereby initiating pyroptosis 106, 111. In the non-canonical inflammasome activation pathway, inflammasomes are not involved, however caspase-4 and caspase-5 in humans or caspase-11 in mice are activated by LPS stimulation or other signals 112. Subsequently, activated caspase-4/5/11 facilitates the formation of active GSDMD N-terminal fragment (GSDMD-N) which binds to acidic phospholipids on the plasma membrane and induces oligomeric pores, leading to cytolysis and activating the pyroptosis cascade 90, 113, 114. Aside from the two common pathways mentioned above, an incomplete pyroptosis has been observed in target cells like macrophages lacking caspase-1 and caspase-11 98. The designation "incomplete" refers to the scenario where the pyroptotic process triggers the release of IL-1α rather than the pivotal pyroptosis factor, IL-1β.

Radiotherapy robustly activates the NLRP3 and AIM2 inflammasomes subsequent to cytosolic DNA production, not only facilitating the secretion of IL-1β and IL-18 but also readying the cellular machinery for pyroptotic cell death following GSDMD cleavage by inflammatory caspases1/3/4 111, 115. Besides, accumulating evidence elucidates that irradiation can directly trigger pyroptosis in diverse tumors through GSDME-mediated pathway. In nasopharyngeal carcinoma (NPC), Zhao C et al. reported that OTUD4 deubiquitinated and stabilized GSDME, enhancing radiosensitivity of NPC cells by promoting their pyroptosis 116. The proportion of pyroptotic cells, and the cleavage of GSDME increased in a dose- and time-dependent manner. In other words, increasing doses of irradiation has a positive correlation to pyroptosis activity in NPC cells. In colorectal cancer (CRC), irradiation can induce GSDME-mediated pyroptosis and overexpressing GSDME sensitizes CRC cells to irradiation and increases NK cells infiltration in TME to enhance anti-tumor immunity 117. In addition, Cao W et al. revealed that irradiation can induce pyroptosis in GSDME high-expressing tumor cell lines covering lung, liver, breast, and glioma cancers 118. To summarize, the GSDME expression in tumor cells is a predictor of radiosensitivity and conferred radiotherapy prognosis in various tumors.

Aside from inducing pyroptosis in tumor cells, irradiation can also stimulate immune cells to develop pyroptosis. Both resident and bone marrow-derived macrophages (BMDMs) exhibit high sensitivity to pyroptosis induction upon exposure to radiotherapy 107, 119. Liu YG et al. observed an elevating caspase-1 activation and an increasing pyroptotic level of bone marrow-derived macrophages in 10Gy and 20Gy radiation groups 119. Furthermore, pyroptosis of immune cells driven by irradiation can activate anti-tumor immunity by releasing inflammatory cytokines, therefore enhancing the efficacy of radiotherapy. Han CH et al. revealed that irradiation-induced AIM2 or NLRP3 inflammasome activation in macrophages can trigger IL-1 signaling pathway to conduct IL-1α release, which activates anti-tumor immunity and further improves the efficacy of radiotherapy 120.

Radiation-induced pyroptosis not only facilitates the immunogenic cell death of tumors but also leads to significant tissue damage. Pyroptosis has been identified as a prominent factor in radiation-induced lung injury (RILI) and radiation-induced intestine injury (RIII) 121. Gao J et al. illustrated that suppression of AIM2-mediated pyroptosis of BMDM can greatly ameliorate RILI and lung fibrosis in C57BL/6 mice when exposed to radiation doses of 18Gy 122. Likewise, a study conducted by Tan G et al. demonstrated that GSDME amplifies the extent of radiation-induced tissue damage, affecting organs such as the intestine, stomach, liver, and pancreas through the escalation of pyroptosis in epithelial cells 117. These findings strongly imply the relevance of pyroptosis and radiation-induced tissue injury.

### Irradiation and Ferroptosis

In 2012, Stockwell BR et al. defined a unique form of programmed cell death, dependent on intracellular iron, coined ferroptosis 123, 124. Ferroptosis is driven by the accumulation of lipid peroxidases, resulting in the rupture of the plasma membrane. Excessive intracellular iron contributes to lipid peroxidation by producing reactive oxygen species (ROS) and activating iron-containing enzymes, such as arachidonic acid lipoxygenases (ALOXs) 125-127. In the presence of long-chain fatty acid-CoA ligase4 (ACSL4) and Lysophosphatidylcholine Acyltransferase 3 (LPCAT3), polyunsaturated fatty acid (PUFA) is catalyzed to develop phospholipids-polyunsaturated fatty acid (PL-PUFA) 128-130. Subsequently, ALOXs or Cytochrome P450 Oxidoreductase (POR) activate PL-PUFA to generate phospholipid hydroperoxides (PL-PUFA-OOH), attacking cell membrane to trigger ferroptosis 131.

The gambling between intracellular oxidative and antioxidative process determines whether ferroptosis is to occur. To be more specific, when activity of antioxidative system attenuates and oxidative system enhances, ferroptosis is more likely to occur 132. The failure of antioxidant process is attributed to the glutathione depletion and the decrease in the synthesis of cysteine/glutamate transporting system Xc^-^, composed of SLC7A11 and SLC3A2. Additionally, loss of glutathione causes decreased synthesis of glutathione peroxidase 4 (GPX4), so that lipid oxides can no longer be metabolized 133-135. Dysfunction of other antioxidative defense systems, such as the coenzyme apoptosis-inducing factor mitochondrial 2-coenzyme Q10 (AIFM2-Q10), tetrahydrobiopterin (BH), as well as endosomal sorting complex required for transport III (ESCRT-III) membrane repair system all add to ferroptosis occurrence 136-138.

Accumulating evidences have proved the relevance between irradiation and ferroptosis. After irradiation, tumor cells exhibit increased expression of ferroptosis marker, gene prostaglandin peroxide synthase 2 (PTGS2), as well as modifications in morphological features: (i) rupture and blistering of cell membrane, (ii) wrinkling mitochondria with disappearance of mitochondrial cristae, and (iii) nucleus lacking of chromatin condensation 139-141. In addition, the ferroptosis inhibitors such as ferrostain-1 and liproxstatin-1 or even iron chelator DFO partially restored survival following irradiation in diverse tumors. Significantly, the impact of ferroptosis inhibitors was comparable to, or in some cases even more pronounced than, that of other inhibitors targeting apoptosis or necrosis in terms of their ability to mitigate irradiation-induced lethality. This underscores the notion that ferroptosis stands as a prominent form of cell death driven by irradiation 139.

Mechanistically, irradiation may promote ferroptosis through at least three pathways 142. Initially, radiation induces lipid peroxidation by generating excessive ROS. More specifically, ROS created by radiation can extract electrons from PUFAs to form PUFA radicals and subsequently interact with oxygen molecules to produce lipid peroxyl radicals (PUFA-OO), which eventually produce lipid peroxides through the Fenton reaction 143, 144. Secondly, radiation promotes the biosynthesis of PUFA-PLs by upregulating the expression of ACSL4 139. Gan BY et al. and their colleagues elucidated that ACSL4 knockout or liproxstatin-1 treatment with irradiation could significantly restore tumor growth on C57BL/6 mice. Intriguingly, combinatory treatments of irradiation and ACSL4 KO or pairing liproxstatin-1 effectively reduced occurrence of ferroptosis without affecting other impact of irradiation on tumor inhibition 139. Last but not least, radiotherapy also leads to the depletion of GSH, which further facilitates ferroptosis by attenuating the SLC7A11-GSH-GPX4-mediated antioxidative pathways 140, 141. According to Zou WP's et al. work, irradiation-activated ATM suppresses the expression of transporter SLC7A11 in ID8 and B16F10 cell lines, resulting in reduced GSH synthesis, elevated tumor ferroptosis, and improved tumor control 140. In several NSCLC cell lines, researches demonstrated that irradiation also triggers rising expression level of SLC7A11 and GPX4, as an adaptive response to resist ferroptosis, while upon introducing erastin, the state of radioresistance of NSCLC is counteracted, partially achieved due to GPX-4 mediated pathway inhibition 139, 142, 145. Consequently, delving deeper into the correlation between irradiation and ferroptosis is of paramount significance.

## Targeting Cell Death Programs Driven by Irradiation to Strengthen the Impact of *In Situ* Vaccine

### Switch “Non-Immunogenic” to “Immunogenic” Following Irradiation

As the predominant form of cell death driven by irradiation, apoptosis is regarded as less immunogenic 146. The low capability of apoptotic cells to activate immune responses may be attributed to (i) the inefficient release of DAMPs due to the integrity of cell membrane; (ii) the efferocytosis by phagocytes, mainly macrophages; and (iii) small-molecule metabolites of apoptotic cells to mediate anti-inflammatory activities 147. From the beginning, the membrane of apoptotic cells remains intact so that no DAMPs are released to activate immune responses. Moreover, during apoptosis, lysophosphatidylcholine (LysoPC), sphingosine-1-phosphate (S1P), CXCL3 and ATP/UTP are released, functioning as “find-me” signals, to recruit resident macrophages to initiate phagocytosis 148. Accordingly, the externalization of phosphatidylserine (PS) on apoptotic cell surface, caused by caspase activity, becomes the main “eat-me” signal to mediate clearance by phagocytes in a “silent” way 149, 150. Additionally, it was demonstrated that apoptotic cells release AMP rather than ATP, which is later metabolized to adenosine, can stimulate macrophages *via* the A2 adenosine receptor to mediate the upregulation of anti-inflammatory genes, thereby prohibiting any immune response 151. Aside from extracellular aspects, intracellular factors also explain the low “immunogenicity” of apoptotic cells. Caspase-3, one of the major executors of apoptosis, also participates in immunosuppressive effect of apoptotic cells. The degradation of cyclic GMP-AMP synthase (cGAS) and its downstream molecules to inhibit type I IFNs production, the activation of cytosolic calcium-independent phospholipase A2 to generate prostaglandin E2 (PGE2), are helping to demonstrate the immunosuppressive function of caspase-3 during apoptosis 152.

Given the potential interplay between cell death programs driven by irradiation, it is reasonable to augment the immunomodulatory impact of irradiation by transmitting apoptosis to other forms of immunogenic cell death. As is discussed above, the apoptotic and necroptotic pathways are tightly linked through the activity of caspase-8, which is one of the major executors of extrinsic apoptosis, but also prohibits necroptotic signaling through the cleavage of RIPK1 and possibly RIPK3 153-156. Accordingly, inhibitors of caspase-8, for instance pan caspase inhibitor z-VAD-FMK or caspase-8 specific inhibitor z-IETD-FMK, stabilize the necrosome, providing platform for promoting the necroptosis through RIPK3 dependent MLKL phosphorylation 157, 158. Consistently, Yu JM et al. reported that ablation of caspase-8 increased stimulator of interferon genes (STING) pathway activation and MLKL activity, enhancing the anti-tumor effect of irradiation 159. The finding reveals that ZBP1-MLKL-mediated necroptotic signaling maximizes radiation-induced anti-tumor immunity through communications with the intrinsic STING pathway in tumor cells.

Except for necroptosis, it is feasible to transform apoptosis to pyroptosis after taking caspase protease family members into consideration. To begin with, pyroptosis-induced caspase-1 protease cleaves the Bcl-2 family member Bid, leading to the activation of MOMP and downstream apoptotic signaling pathways 160. The function of caspase-8 in pyroptosis is dual-faceted. To be more specific, when caspase-8 activity is inhibited, the cellular machinery can shift towards pyroptosis or necroptosis. In this case, pan-caspase inhibitors provide an opportunity for pyroptosis. However, caspase-8 can also interact with pyroptotic adapter protein ASC, which forms supramolecular oligomers during inflammasome activation 161, 162. From this perspective, promotion of interplay between caspase-8 and ASC apparently lead to caspase-8 dependent activation of pyroptosis under certain circumstances 163, 164. Apart from that, the more fundamental connection between apoptosis and pyroptosis is based on the observation that GSDME, the structurally and functionally homologous with the pyroptosis effector GSDMD, can be cleaved and activated by apoptosis executor caspase-3. Consequently, GSDME can regulate membrane permeability that occurs in the later period of apoptosis, an event recognized as “secondary necrosis” 95, 165. This discovery indicates that apoptotic events culminating in GSDME-mediated "secondary necrosis" can be classified as a form of pyroptosis. Inflammasomes, as the upstream signals of pyroptosis, are responsible for the activation of inflammatory caspases for instance caspase-1. In certain scenarios, when confronted with both apoptotic and pyroptotic stimuli, activating inflammasomes can take precedence, leading to pyroptosis. Recently, Hou JW et al. illustrated that nuclear PD-L1 can regulate a GSDMC-caspase-8 mediated non-canonical pyroptosis pathway. During this process, PD-L1 nuclear translocation upregulates the transcription of GSDMC in tumor cells, converting apoptosis, induced by TNF-α, into pyroptosis 166. This presents a potential avenue for immunotherapy, specifically targeting PD-L1 blockade, in conjunction with irradiation-induced pyroptosis.

The components involved in the DNA damage response (DDR) process, such as ATM, FANCD2, and BAP1, play significant roles in regulating the onset of ferroptosis. Research has demonstrated that FANCD2 protects bone marrow stromal cells (BMSCs) against ferroptosis. FANCD2-deficient BMSCs display increased vulnerability to erastin-induced ferroptosis 167. FANCD2 influences an array of proteins participating in iron metabolism and lipid peroxidation, notably GPX4, whose inhibition results in the prevention of ferroptosis. Conversely, the oncogene BRCA associated protein 1 (BAP1) has been identified as a positive regulator of ferroptosis. SLC7A11, a target gene downstream of BAP1, accelerates ferroptosis by diminishing SLC7A11 expression *via* a deubiquitination mechanism 168. It has been observed that HMGB1 is released during the onset of ferroptosis and relies on an intact autophagy process. The restraint of HDAC, mediated by autophagy, fosters the acetylation of HMGB1, precipitating the discharge of HMGB1 in response to ferroptosis 169. According to the evidence presented, it is conceivable that aiming at pivotal components of the radiation-induced DDR process can amplify the incidence of ferroptosis and its immunogenicity. Furthermore, by intervening in the aforementioned pathways, there exists potential to facilitate the transition from apoptotic cell death driven by irradiation to ferroptosis.

The immunogenicity of radiation-induced cell death pathways is partially dose dependent. Radiation dose and fraction can be correlated to a specific cell death pathway. Encouse BG et al. demonstrated, escalating doses of radiation reaching up to 20Gy, augmented the release of immunogenic substances like HMGB1 and ATP. Additionally, there was a notable rise in the quantity of CRT anchored to the cell membrane 170. In addition, some studies have revealed that ablative fractionation radiotherapy is more likely to induce necrotizing apoptosis of tumor cells, both *in vivo* and *in vitro* 171, 172. Based on these findings, we can conclude that variation in radiation doses and fractions can modulate cell death pathways and improve tumor immunogenicity. Intensity-modulated radiotherapy (IMRT) and stereotactic radiotherapy (SBRT) allow for treatment with high radiation doses while avoiding damage to the surrounding tissues. Leveraging these advanced technologies and the capability to safely administer radiation at elevated doses allows us to evoke a robust immunogenic cell death response while preserving adjacent tissues.

For decades, efforts have been dedicated toward the development of radiosensitizers. Conventionally, these agents aimed to elevate intrinsic radiosensitivity of malignant cells by augmenting the apoptotic rate. However, recent research has revealed diverse irradiation-induced cell death modes, moving beyond the narrow confines of apoptosis alone. This broader understanding, combined with the heightened role of immune cells post-irradiation, signals a paradigm shift. It indicates that steering the immunological consequences of irradiation-induced cell death might offer more therapeutic promise than simply boosting tumor cell apoptosis. Transitioning from the conventional, non-immunogenic apoptosis to other immunogenic cell death forms enhances the *in situ* vaccine impact initiated by irradiation, thereby potentiating both localized and systemic immune responses.

The transition from apoptosis to ICD post-radiation is influenced by caspases activity. Specifically, the shift from apoptosis to necrotic apoptosis can be modulated by regulating caspase-8 and ZBP1. In contrast, the conversion from apoptosis to pyroptosis is achieved through caspase-3 or caspase-8, overexpression of GSDMs protein, or the application of pan-apoptotic inhibitors. A challenge that arises is the absence or low expression of the GSDMs protein in some tumors, hindering the transformation from apoptosis to pyroptosis. For such GSDMs-deficient tumors, our strategy will give preference to driving apoptosis towards necroptosis mediated by caspase activity, instead of pyroptosis. Meanwhile, the promotion of a transition from apoptosis to ferroptosis remains to be further evidenced. Ferroptosis appears to operate independently of the caspase machinery, primarily *via* iron-mediated lipid peroxidation. However, it remains to be determined whether ferroptosis can be linked to other cell death modalities through caspases. Additionally, the specific definition of immunogenicity and the profile of immunogenic markers released during ferroptosis still warrant further investigation.

## The Synergistic Impact of Radiotherapy Combining with Immunotherapy Greatly Modulates Cell Death Pathways and Reinforces *In Situ* Vaccination

With the advent of immune checkpoint inhibitors (ICIs), immunotherapy has been one of the most powerful weapons for cancer treatment. Irradiation can elicit potent anti-tumor immune response by influencing almost all processes in the tumor-immunity cycle rather than merely several discrete steps with ICIs 173, 174. These effects encompass the augmented release and presentation of tumor-derived antigens, improved recognition of tumor cells by T cells, stimulation of immune cells initiation and activation, and an elevated count of tumor-infiltrating lymphocytes. Together, these mechanisms reinforce the anti-tumor response 175-178. Beyond the aforementioned effects, irradiation also leads to the release of pro-inflammatory cytokines *via* the intracellular cGAS-STING pathway and other inflammatory signaling pathways 179. From this perspective, irradiation induces a remodeling of the tumor microenvironment through inflammatory cytokines, vascular changes and immunological components, collectively facilitating systemic anti-tumor responses 180, 181. This newly-irradiation-induced reprogrammed tumor microenvironment is a revolutionary discovery, as we can potentially transform “cold” tumors, with fewer immune cells, into “hot” tumors with more lymphocytic infiltration, providing a foundation for effective response to ICIs 182.

### The Rationale of Combining Irradiation-induced *In Situ* Vaccines with Immunotherapies to Achieve Anti-tumor Treatment

Previously, it was regarded that DAMPs and TAAs released by tumor cells following irradiation facilitated the generation of *in situ* vaccines. However, as our understanding of immunomodulatory effects driven by irradiation has deepened, it becomes obvious that various factors contribute to this *in situ* vaccine impact, including pro-inflammatory cytokines released by tumor cells, intrinsic immune activation pathways of tumor cells, neoantigens presented by the tumor cells during irradiation, and responses of immune cells within the tumor microenvironment after being irradiated. Modulating these elements can reinforce the *in situ* vaccine impact, potentiating synergistic outcomes when in combination with immunotherapy.

#### Cytokines Release

Contemporary research examining the interplay between radiation and immunity have unequivocally highlighted the pivotal role cytokines assume in the activation of systemic anti-tumor immunity *via* radiotherapy 183. For instance, irradiation-induced GM-CSF guarantees the advancement of the maturation of DCs. GM-CSF enables DCs to efficiently capture tumor antigens, present them to adaptive immune cells, and trigger the ensuing anti-tumor immune response 184. Similar to GM-CSF, another innate immunity associated cytokine M-CSF is renowned for promoting maturation and differentiation of macrophages, its release driven by irradiation often exhibits immunosuppressive impact. To elaborate, M-CSF driven by irradiation can result in the accumulation of tumor associated macrophages (TAMs) and myeloid-derived suppressor cells (MDSCs) within the tumor microenvironment. Both of these entities release anti-inflammatory cytokines that impede anti-tumor immunity. Clinical investigations by Autio KA et al. revealed that the M-CSF receptor inhibition with antibody LY3022855, offers positive immunomodulation in patients with advanced breast or prostate cancer 185. While M-CSF often facilitates immunosuppression, its combination with ICIs could counteract these effects, bolstering anti-tumor immune responses. Consequently, the precise role of M-CSF in the nexus of radiotherapy and immunotherapy warrants deeper investigation. Cytokines CXCL9 and CXCL10, when released from tumor cells following irradiation, can drive T cell chemotaxis, facilitating their migration into the tumor microenvironment. Several investigations indicate that irradiation stimulates tumor cells to release CXCL16 which can interact with Th1 cells and CXCR6 present on activated CD8^+^ T cells, leading to enhanced local infiltration of immune cells 186. Furthermore, radiotherapy can prompt an upregulation of IFN-γ related gene transcription in tumor cells, leading to IFN-γ secretion that bolsters T cell effector functions. IL-2, pivotal for supporting T cell proliferation and survival, is often termed T cells' "third signal." Preliminary research has revealed that marrying radiotherapy with high-dose IL-2 produces a synergistic effect, enhancing the immune response in a murine adenocarcinoma model with low immunogenicity 187. Numerous ongoing studies aim to optimize IL-2 delivery or release systems to elevate its concentration within the tumor microenvironment to augment immunotherapy efficacy 188. Besides IL-2, cytokines like IL-3, IL-12, and TNF-α have also been appraised in preclinical studies alongside radiotherapy, demonstrating their potential in enhancing radiation-induced immune responses 189.

Of note, the radiation-induced transforming growth factor β (TGF-β) originated from the extracellular matrix stands out as a key cytokine that fosters immunosuppressive tumor microenvironment 190. Elevated TGF-β results in the poor prognosis, by inducing epithelial-to-mesenchymal transition (EMT) in tumor cells, recruiting immunosuppressive cells to TME and hampering CD8^+^ T cells function 191. In triple-negative breast cancer (TNBC), TGF-β has also been implicated in boosting the expression of PD-L1, a mechanism by which tumors evade immune surveillance 192, 193. The blockade of TGF-β during radiotherapy, for instance application of Tranilast, has proven effective in stimulating specific CD8^+^ T cell responses against native tumor antigens 193. Concurrently, when immunotherapy is administered alongside radiotherapy, it can partially counteract the immune suppression resulting from the TGF-β-mediated increase in PD-L1 expression.

#### cGAS-STING Pathway Stimulation

It is well established that radiation-induced DDR affects immunological responsiveness of irradiated cells. A noteworthy manifestation of this immunogenicity is the activation of the cGAS-STING signaling pathway within tumor cells, leading to type I IFNs production. cGAS serves as an interface linking the DDR to STING signaling initiation. The DNA that cGAS receptors recognize encompass micronuclei containing DNA, which enters the cytoplasm after radiation, and damaged mtDNA. They oligomerize with cGAS in the form of a complex, facilitating the synthesis of 2'3'-cyclic GMP-AMP (cGAMP) which in turn activates STING, promoting its movement to the Golgi apparatus, triggering TANK-binding kinase 1 (TBK1) 194-197. Subsequently, TBK1 phosphorylates STING, driving the translocation of interferon regulatory factor 3 (IRF3) to the nucleus and initiating the transcription of type I IFN genes 198, 199. The type I IFNs driven by activation of cGAS-STING signaling pathway play a pivotal role in immune modulation for it not only amplifies the antigen-presenting function but also boosts CD8^+^ T cells activity, supporting anti-tumor immunity. While, on one hand, the system requires type I IFN signaling to initiate adaptive immunity, leading to tumor rejection; on the other hand, prolonged type I IFN signaling can prompt effector T cells to express inhibitory transcription factors, initiating their exhaustion 200, 201. Thus, from this vantage point, during extended radiotherapy, infiltrating T cells in the tumor microenvironment might exhibit exhausted state. The introduction of Immune Checkpoint Blockades (ICB) offers an opportunity to counteract this T cell exhaustion and reboot the effector program. Moreover, there is evidence suggesting that the response patterns of a patient's preoperative peripheral blood type I IFN can forecast the therapeutic efficacy of PD-1 monoclonal antibodies (McAb) 202. Fan J et al. declared that pairing IFN-α with anti-PD-1 develop obvious tumor inhibition 203. This substantiates the logic behind combining radiotherapy with immunotherapy.

#### Neoantigens Presentation

Beyond the release of DAMPs and pro-inflammatory cytokines derived from ICD, irradiation can also lead to neoantigens production. It is widely acknowledged that tumor cells initiate DDR to cope with DNA damage driven by irradiation. However, disorders in the DDR processes are frequently observed in tumors, potentially leading to persistent genomic instability. As this instability piles up, it gives rise to genomic mutations. These mutations, in turn, lead to the synthesis of novel proteins, commonly referred to as neoantigens. Radiotherapy raises the expression level of MHC-I in tumor cells, thereby enhancing the presentation of neoantigens on the tumor cell surface. Reits has elucidated that within the initial 4 hours post-irradiation, free radicals hasten the degradation of intracellular proteins and activate the mTOR signaling pathway, subsequently augmenting the synthesis of corresponding proteins 204. These newly synthesized proteins are then loaded onto MHC-I molecules, leading to the display of new peptides on the MHC-I molecules, peptides that are absent in non-irradiated cells. And a recent study by Lussier DM et al. confirmed that tumors with low mutation burden and poor antigenicity can acquire neoantigens through accumulated mutations driven by irradiation, resulting in the improved efficacy of immunotherapy 205. These studies unveil an additional potential intervention to maximize treatment efficacy by targeting irradiation-generated neoantigens.

#### Immune Configuration Modification

The impact of radiotherapy on T cells has attracted the focus of numerous investigations. Notably, T cell subpopulations display varied sensitivities to radiation. It is widely acknowledged that CD4^+^ T cells exhibit greater resilience to radiation compared to CD8^+^ T cells. Remarkably, among the CD4^+^ T cells, the regulatory T (Treg) cells, characterized by their Foxp3 expression, exhibit even more radioresistance 206, 207. Several studies have highlighted that radiation therapy augments the population of Treg cells infiltrating the tumor microenvironment (TME) 208. Muroyama Y et al. documented that radiotherapy strengthens the immunosuppressive capabilities of Treg cells within the TME 209. In parallel, research by Owedia discerned that STAT3, a principal regulator of Foxp3, promotes the radiation-induced transmission of infiltrating CD4^+^ T cells towards Treg cells. This STAT3 activation is steered by the binding interaction between IL-10 and its receptor. Such an interaction catalyzes Treg cell differentiation, marked by a spike in CTLA-4 expression levels, intensifying the immunosuppressive milieu 210. Given these findings, a combinatory approach of radiotherapy and CTLA-4 monoclonal antibody-based immunotherapy is justifiable when viewed through the lens of Treg cell dynamics. It's noteworthy that while high-dose radiation bolsters the presentation of MHC-I molecules, DAMPs, and TAAs, facilitating T-cell activation and infiltration, it concurrently inflicts direct damage on T cells. Contrarily, while low-dose radiation may lack the potency to eliminate tumor cells, it can stimulate immune cell activation, ameliorate the stromal microenvironment, fortify the systemic immune response, thereby reinforcing the effects of immunotherapy 211, 212. Hence, the interplay between T cells and radiation is intricate, with scenarios where irradiation might attenuate T-cell-mediated adaptive immunity. Yet, integrating immunotherapy with radiotherapy can potentially tip the immune balance towards therapeutic benefit.

Beyond its impact on T cells, radiation significantly influences other immune cells. For instance, Zhu B et al. observed that radiation amplifies the infiltration of eosinophils within tumors. Intriguingly, T cell infiltration diminished with eosinophil depletion, attenuating the anti-tumor immune effects induced by irradiation. Furthermore, the administration of IL-5 boosts eosinophil activation, augmenting the abscopal effect 213. Together, eosinophil mobilization driven by irradiation may serve as a conduit, bolstering CD8^+^ T cell-mediated anti-tumor immunity and improving T cell-centered immunotherapy efficacy. Natural Killer (NK) cells, intrinsic components of the immune system, primarily combat infectious and malignant cells. Their major cytotoxic mechanisms include the release of granzyme B and perforin, in addition to facilitating antibody-dependent cellular cytotoxicity (ADCC) effects. While NK cell-based immunotherapies are under intensive research, the precise role of NK cells within the confluence of combined radiotherapy-immunotherapy remains ambiguous 214. In a study involving a canine sarcoma model, a notable escalation in NK cell homing to tumor sites was observed following radiotherapy 215. This coincided with augmented cytotoxicity and enhanced activation of circulating NK cells, culminating in tumor regression. The therapeutic response of NK cells might be augmented by radiation-induced elevation in the expression of NKG2D ligand-NKG2DLs, which could further stimulate NK cells and CAR-NK cells, ascribed to NKG2D editing 216. It's also pertinent to note that the function of NK cells is significantly influenced by numerous immune checkpoints, including TIGIT, PD-1, and killer immunoglobulin-like receptors (KIR) 217, 218. Utilizing ICIs to block these checkpoints can alleviate the functional constraints on NK cells 219. Accordingly, by engaging with NK cells, radiotherapy has the potential to amplify the benefits of immunotherapy.

### Different Cell Death Pathway Signals Sensitize Immunotherapy

As we delve deeper into the synergistic mechanisms surrounding radiotherapy and immunotherapy, the relationships between the various cell death pathways driven by both treatments are being actively investigated. For instance, pyroptosis of tumor cells can enhance anti-tumor immunity. Shao F et al. and their colleagues applied an innovatively designed biochemical method to specifically engage tumor cells. This drug was delivered to mice that overexpressed GSDM family proteins. While pyroptosis was detected in only approximately 15% of tumor cells, it was capable of triggering a strong anti-tumor immune response, resulting in nearly complete tumor clearance 220. These data suggest that inflammation caused by pyroptosis triggers a strong anti-tumor immunity response and together with ICIs achieves a synergistic effect. At the same time, Judy Lieberman's team showed similar results: the cell-killing enzyme Granzyme B can directly cleave GSDME, causing pyroptosis of cancer cells, further activating the anti-tumor immune response and inhibiting tumor growth 99. Together, this concludes that pyroptosis can indeed turn a "cold" tumor into a "hot" tumor, enhancing the efficacy of ICIs.

In addition to pyroptosis, studies have also demonstrated that the synergistic effect of radiotherapy and immunotherapy is connected to the heightened activity of ferroptosis. *In vitro*, DAMPs released from tumor cells undergoing ferroptosis trigger pro-inflammatory responses, inciting the maturation of dendritic cells, cross-priming of CD8^+^ T cells, and reprogramming of M2-type macrophages to M1-type. To be more specific, lipid peroxidation associated with ferroptosis can serve as a "find me" signal facilitating the recognition, phagocytosis and presentation of tumor antigens by DCs, which activates CD8^+^ cytotoxic T cells, promotes the release of IFN-γ, and consequently enhances anti-tumor immunity 221, 222. These alterations enable tumors to adapt to the tumor microenvironment and establish a positive feedback loop of immune responses. In reaction to immune checkpoint inhibitors, such as PD-L1 and CTLA-4, CD8^+^ T cells impede tumor cell cystine uptake and bolster the lipid peroxidation process by downregulating SLC3A2 and SLC711 *via* the release of IFN-γ, while augmenting T cell-mediated anti-tumor immunity and inducing ferroptosis in tumor cells 223, 224. On the one hand, radiation-induced ROS generation and ACSL4 upregulation intensify lipid peroxidation process, leading to subsequent membrane rupture and initiation of ferroptosis. On the other hand, immune checkpoint inhibitors, for instance anti-PD-1 and anti-PD-L1, can suppress the expression level of SLC7A11 and GPX4 on tumors, activate CD8^+^ T cells in tumor microenvironment, and enhance the release of IFN-γ. Therefore, the combined treatment of radiotherapy and immunotherapy robustly elicits ferroptosis and guarantee further anti-tumor immune responses through the occurrence of *in situ* vaccines.

## Conclusions and Perspectives

Radiotherapy acts as a neoadjuvant for immunomodulation, inciting *in situ* vaccine through the release of DAMPs, TAAs, and pro-inflammatory cytokines. Nevertheless, TME after irradiation is remarkably intricate. To boost radiation-induced immunogenicity in support of local or systemic immune responses, various strategies are being taken into consideration. From the perspective of enhancing antigen presentation within the tumor microenvironment, augmenting the functionality of dedicated antigen-presenting cells, particularly DCs, is a prudent strategy. Administration of Flt3L, an agent typically employed to expand dendritic cells, augments dendritic cells antigen presentation capabilities. Combined treatment with radiotherapy and Flt3L exhibited reduced lung metastasis in a Lewis lung carcinoma mouse model, and significantly prolonged survival 225. Brody JD et al. and colleagues conceived an *in situ* vaccine merging Flt3L, radiotherapy, and a TLR3 agonist. This was designed to mobilize, load antigens, and stimulate intratumoral cross-presenting dendritic cells. Their clinical trial NCT01976585 revealed that the *in situ* vaccine elicited anti-tumor CD8^+^ T cell responses and systemically mitigated tumors in patients with advanced iNHL 226. From the perspective of facilitating antigen release during irradiation for recognition by antigen-presenting cells, the exploration of neoantigens is currently a prominent area of research. Huang KC et al. crafted an adenoviral-vectored tumor neoantigen vaccine that overcomes the inhibition of the immune checkpoint inhibitor PD-1/PD-L1 monoclonal antibody on DCs by TLR9 inhibitory fragments, PD-1 trap, and PD-L1 miRNA, resulting in stronger antigen presentation and long-lasting neoantigen-specific cytotoxic lymphocyte responses 227. From the perspective of alleviating inhibitory factors in the post-radiation tumor immune microenvironment, TGF-β serves as one of the crucial regulators. Radiation can instigate local immunosuppression and T-cell depletion by prompting the release of TGF-β from the extracellular matrix, while TGF-β inhibition can counter this effect 193. With regards to newly booming immunotherapy such as CAR-T cell therapy, radiotherapy is also proving to be an indispensable ally. In a pancreatic cancer model exhibiting heterogeneous sLeA expression, a blend of low-dose radiation and CAR-T cell therapy heightened the susceptibility of non-target sLeA negative cells to CAR-T cells 228. The synergy of local radiotherapy and NKG2D edited-based T-cell therapy has shown parallel outcomes 216. The amalgamation of above interventions alongside radiotherapy to augment anti-tumor therapy efficacy has several inherent challenges. The primary concern is the potential for toxic side effects. Several cytokine inhibitors or targeted McAbs have exhibited non-negligible toxicities, as illustrated by the discontinuation of the CD47 McAb Magrolimab study due to hematologic toxicity. Additionally, combining these with radiotherapy could elevate the risk of toxicity. A further constraint hampering the clinical transition of these combined strategies is the disparity between animal models and the real-world clinical scenarios, repelling model translation from experimental animals to patients.

While these strategies stem from diverse perspectives, the fundamental rationale for irradiation-induced reshaping of the immune landscape within the tumor microenvironment (TME) is primarily associated with the balance between cytoprotective signals and cytotoxic signals. With damage signals piled up, multiple cell death pathways are initiated. Hence, fine-tuning the immunological outcome spurred by irradiation-driven cell death signals might be an optimal approach to harness radiation *in situ* vaccine potential. This review delves into the impact of irradiation on various cell death pathways and their interaction with the immune system to stimulate anti-tumor immunity. Additionally, strategies for the optimization of an *in situ* vaccine impact by exploiting these various cell death pathways are also analyzed in this review.

In recent years, new forms of cell death pathways have been revealed, broadening the horizon of cell death programs 229. The cell death pathways driven by radiotherapy should not be limited to apoptosis, necroptosis, pyroptosis or ferroptosis, which were mentioned above. The potential of radiotherapy to instigate other types of cell death, including parthanatos, alkaliptosis, or oxeiptosis, and the subsequent implications for systemic immunity warrant further investigation 230. Initial researches sought to emphasize the distinct regulation of each cell death pathway, but emerging researches have revealed the intrinsic connections and crosstalk between these seemingly unrelated modes of cell death, leading to the concept of PANoptosis 231. The multiple findings mentioned in the review suggest that radiotherapy-induced cell death pathways can hardly be encapsulated into a single cell death form, supporting the idea of PANoptosis occurrence after irradiation. In essence, the pivotal mechanism of PANoptosis relies on ZBP1 activation which initiates the assembly of various signaling complexes to execute multiple cell death programs. And ZBP1 was upregulated after radiotherapy according to research by Yu JM et al. 159. Given this, it's plausible to implicate that the modes of radiation-induced cell death align with PANoptosis. This provides a novel viewpoint for examining the effects of radiotherapy. Exploring how radiotherapy triggers multiple cell death programs can optimize therapeutic outcomes when integrating radiotherapy with immunotherapy.

Taking all into consideration, the cell death-orientated immunomodulatory impacts driven by irradiation remain promising. Significant challenges still exist: (i) the correlation behind radiation doses/fractions to trigger cell death pathways; (ii) the distinct immunogenicity of diverse cell death pathways driven by irradiation and their capacity to activate the local and systemic immunity; (iii) strategies to reinforce the interaction between radiotherapy and immunotherapy by delving into factors that govern differential activation of cell death signals among malignant cells, immune cells and stromal cells following irradiation. As technology in molecular biology advances and preclinical data in support of these notions accumulates, the challenges will be addressed in the next generation.

## Figures and Tables

**Figure 1:**
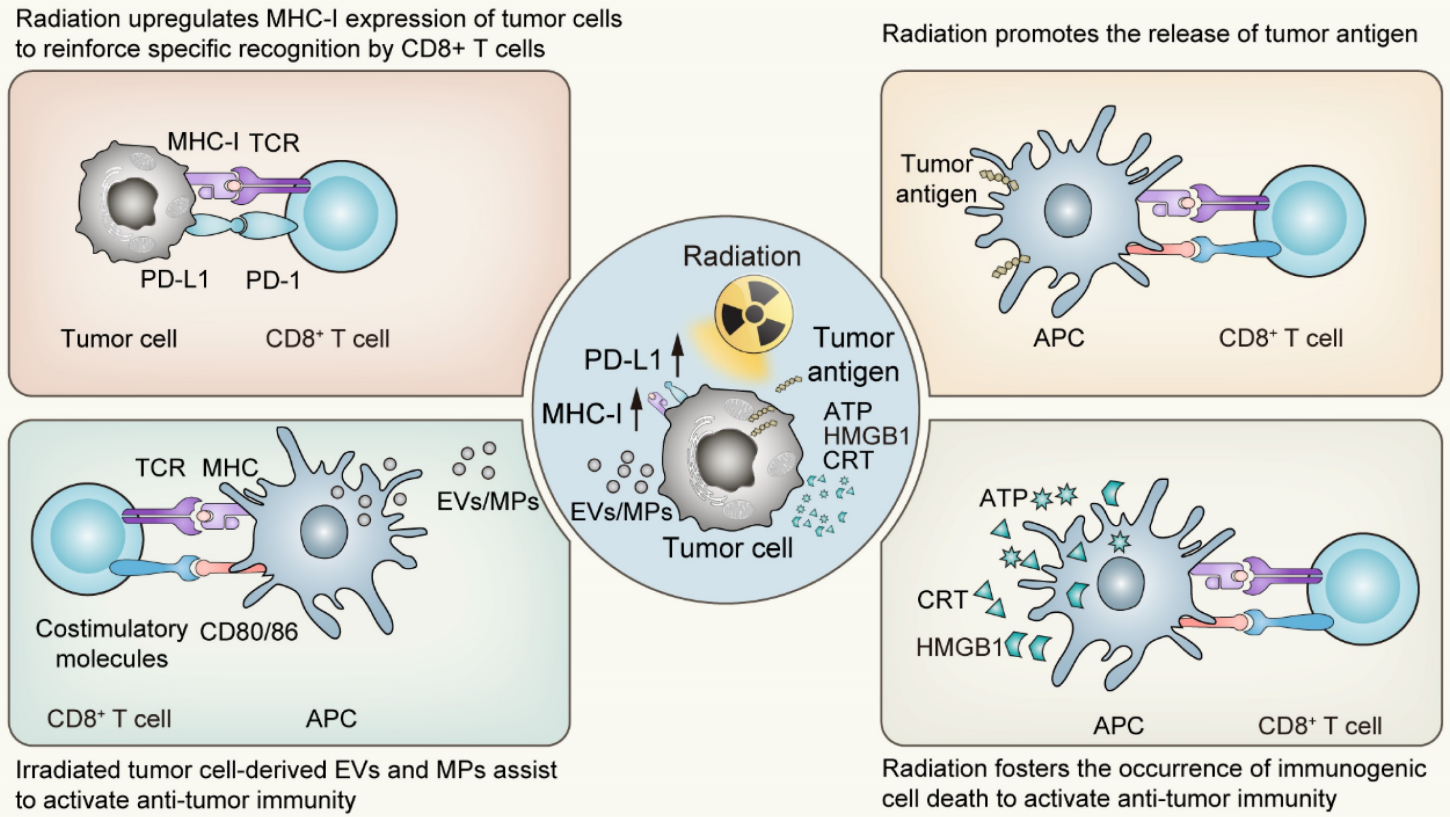
The “*in situ* vaccine” impact triggered by radiotherapy on tumor microenvironment. APC, Antigen-presenting cells; EVs, Extracellular vesicles; MPs, Microparticles; ATP, Adenosine triphosphate; HMGB1, High mobility group box 1; CRT, Calreticulin.

**Figure 2:**
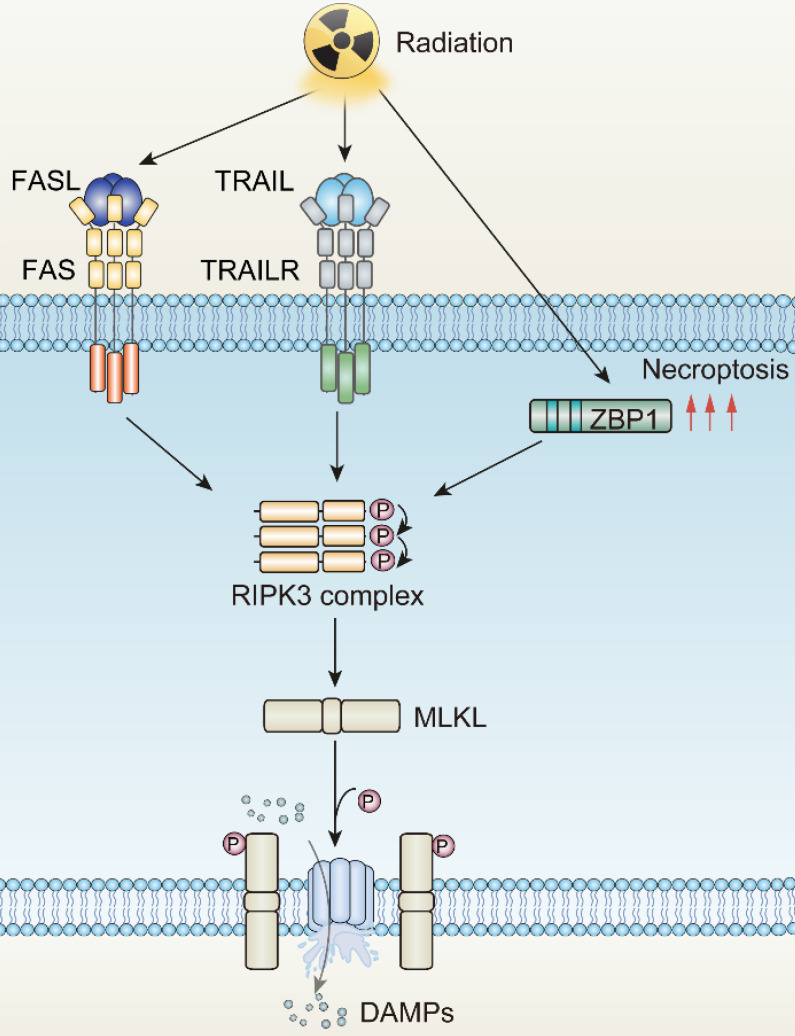
The mechanisms underlying radiotherapy-induced necroptosis. FASL, Fas ligand; TRAIL, Tumor necrosis factor related apoptosis-induced ligand; TRAILR, Tumor necrosis factor related apoptosis-induced ligand receptor; ZBP1, Z-DNA binding protein 1; RIPK3, RIP kinases 3; MLKL, mixed-lineage kinase domain-like protein.

**Figure 3:**
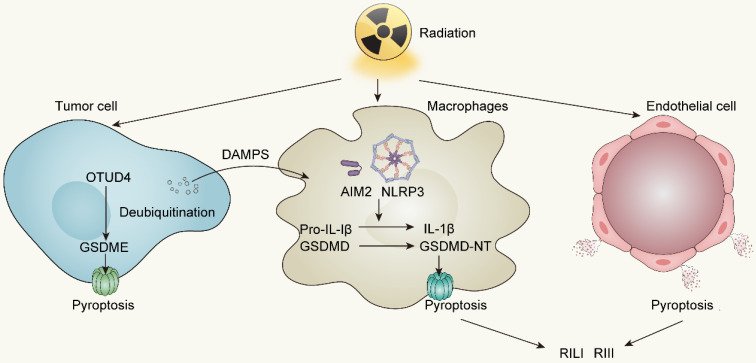
Radiotherapy-induced pyroptosis in tumor cells, immune cells and endothelial cells. OTUD4, OTU deubiquitinase 4; AIM2, Absent in melanoma 2; NLRP3, NOD-like receptor thermal protein domain associated protein 3; RILI, Radiation-induced lung injury; RIII, Radiation-induced intestine injury.

**Figure 4:**
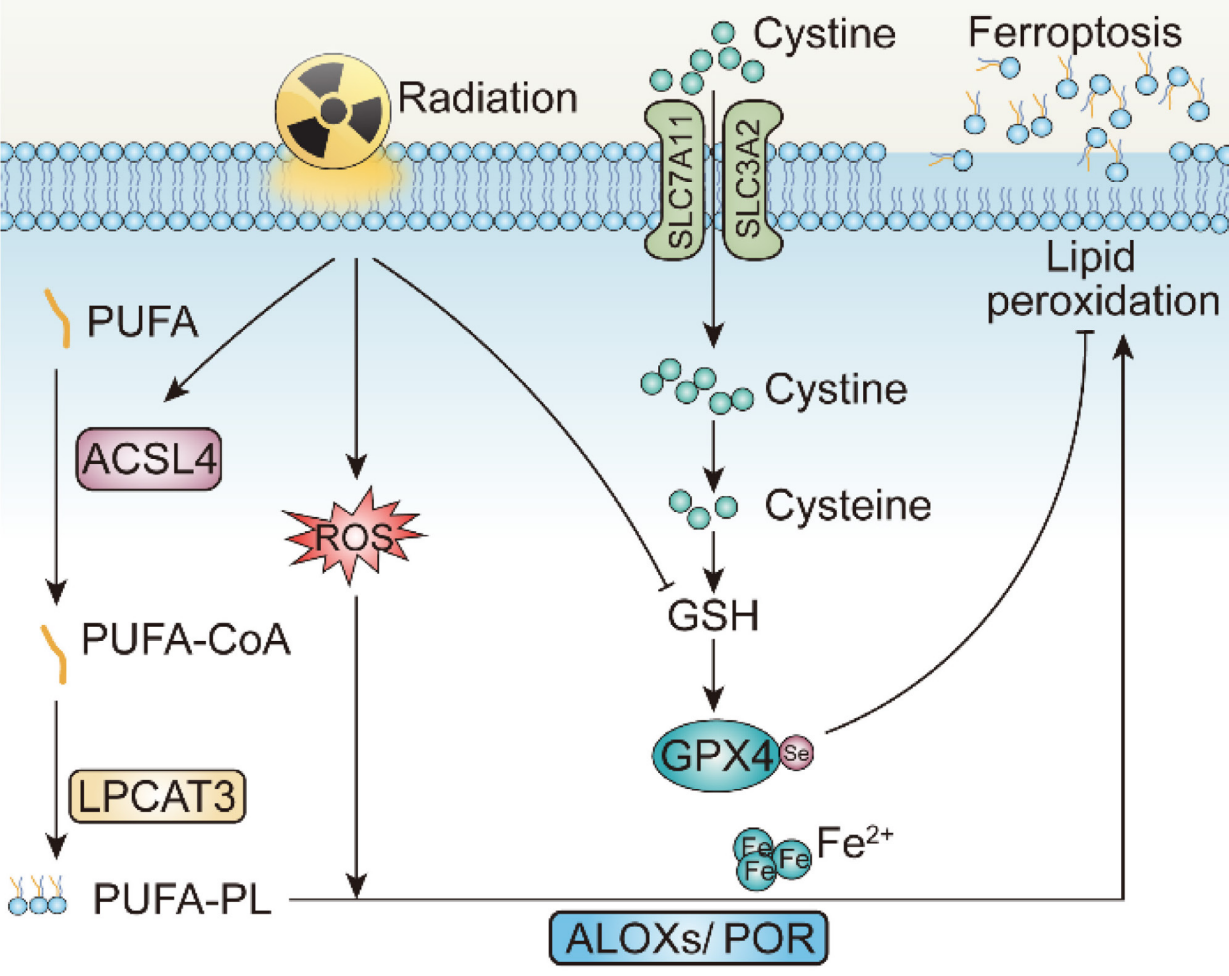
The mechanisms underlying radiotherapy-induced ferroptosis. PUFA, Polyunsaturated fatty acid; ACSL4, Long-chain fatty acid-CoA ligase4; LPCAT3, Lysophosphatidylcholine Acyltransferase 3; GPX4, Glutathione peroxidase 4; ALOXs, Arachidonic acid lipoxygenases; POR, Cytochrome P450 Oxidoreductase.

**Figure 5:**
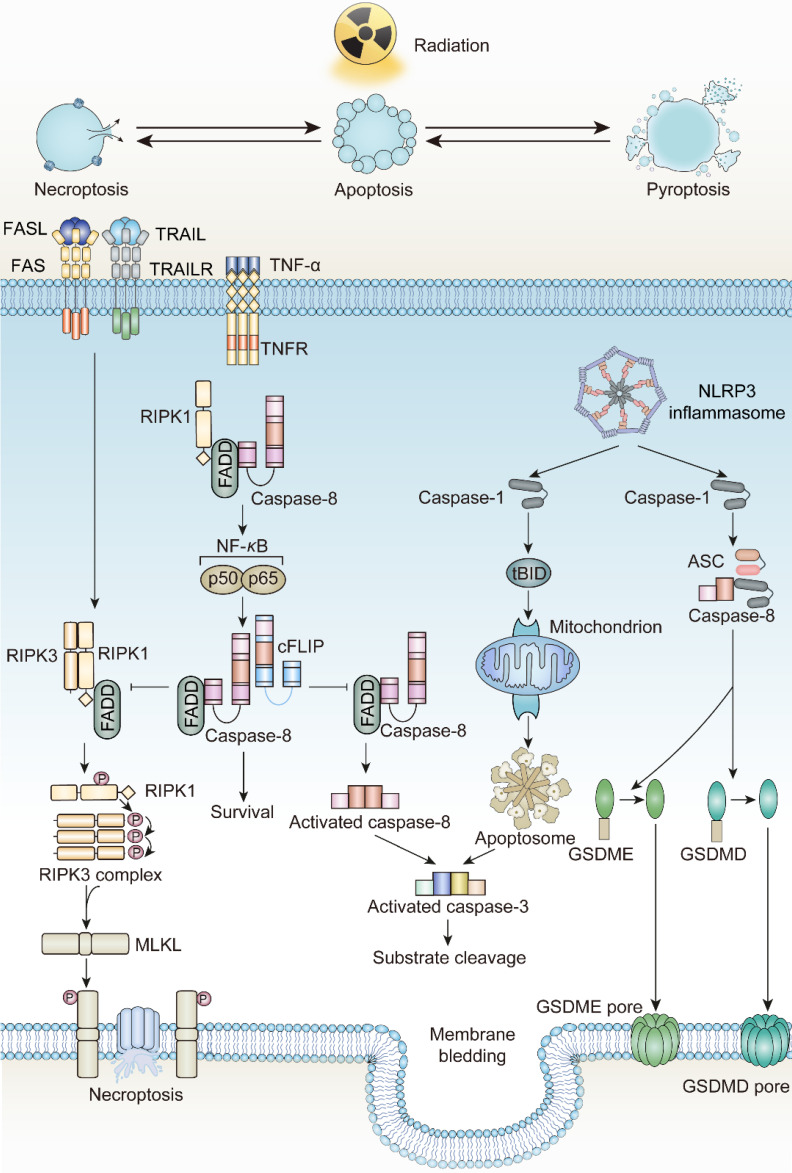
The network interactions between different cell death pathways driven by radiotherapy. FADD, Fas Associated *Via* Death Domain; cFLIP, Cellular FLICE-inhibitory protein; tBID, truncated BID; ASC, Apoptosis-Associated Speck-Like Protein Containing A CARD.

**Figure 6:**
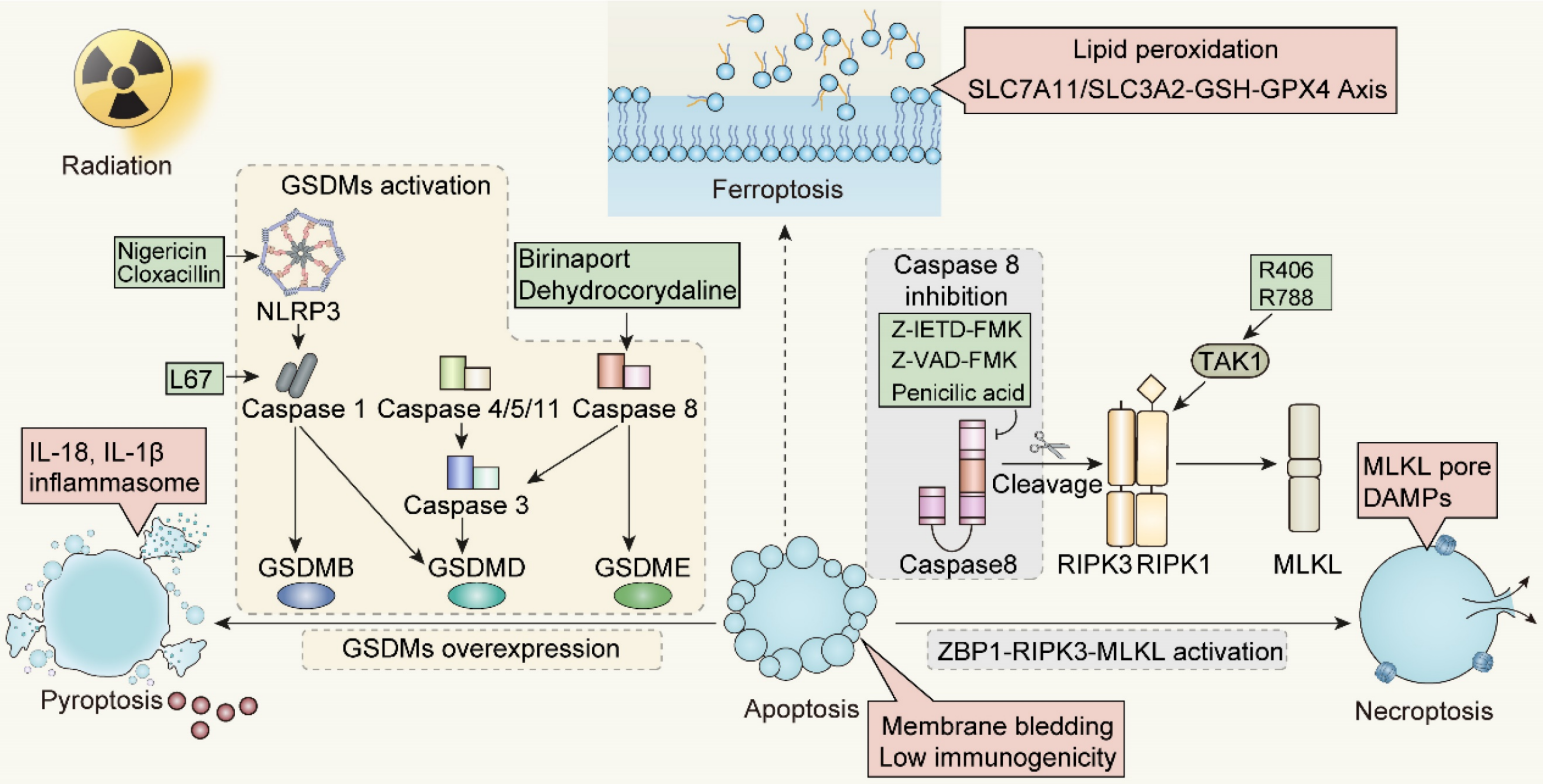
Strategies and potential targets of redirecting apoptosis toward ICD-mediated forms. NLRP3, NOD-like receptor family members 3; TAK1, TGF-β activated kinase 1; RIPK1, Receptor-interacting protein kinases 1; RIPK3, Receptor-interacting protein kinases 3; ZBP1, Z-DNA binding protein 1; MLKL, Mixed-lineage kinase domain-like.
